# Intersecting Anemias: PARP Inhibitor‐Induced Toxicity and Autoimmune Hemolysis—A Case Report

**DOI:** 10.1002/jha2.70112

**Published:** 2025-07-28

**Authors:** Julien Dereme, Konstantinos Asonitis, Francesco Grandoni, Aikaterini Liapi, Apostolos Sarivalasis, Gerasimos Tsilimidos

**Affiliations:** ^1^ Service and Central Laboratory of Haematology Department of Oncology and Department of Laboratories and Pathology Lausanne University Hospital (CHUV) and University of Lausanne (UNIL) Lausanne Switzerland; ^2^ Medical Oncology Unit Department of Oncology Lausanne University Hospital (CHUV) and University of Lausanne (UNIL) Lausanne Switzerland

**Keywords:** hematotoxicity, hemolytic anemia, PARP inhibitors, T‐LGL clone, warm autoantibodies

## Abstract

We present a case of a 71‐year‐old woman with a history of high‐grade serous ovarian adenocarcinoma, treated with neoadjuvant chemotherapy, surgery, and adjuvant chemotherapy. A maintenance treatment combining bevacizumab and olaparib was introduced consecutively. Bevacizumab was stopped 7 months later due to neurological complications. The patient developed severe, progressive anemia, leading to significant clinical management challenges, with hemoglobin levels as low as 54 g/L. Despite initially adapting and finally discontinuing olaparib, the patient remained transfusion dependent, without notable improvement. Common causes of anemia, such as iron deficiency, vitamin deficiency, autoimmune and infectious causes, were ruled out. Bone marrow biopsies revealed a clonal cytotoxic T‐LGL population and a DNMT3A mutation without evidence of myelodysplasia or metastatic infiltration. A further decline in reticulocytes and the appearance of warm autoantibodies (IgG) indicated an increasingly mixed anemia profile. High‐dose corticosteroids resulted in rapid hematological improvement. This case highlights a severe anemia of mixed origin with central hypo‐regenerative components due to prolonged PARP inhibitor toxicity and a hemolytic mechanism associated with warm autoantibodies. The prolonged toxic effect of olaparib, in conjunction with confounding factors (DNMT3A mutation, warm autoantibodies, T‐LGL clone), underscores the need for a comprehensive hematological assessment. The patient's response emphasizes the complexity of managing drug‐induced hematologic toxicity and the potential for overlapping hematologic conditions.

## 1 Case Report

**FIGURE 1:**
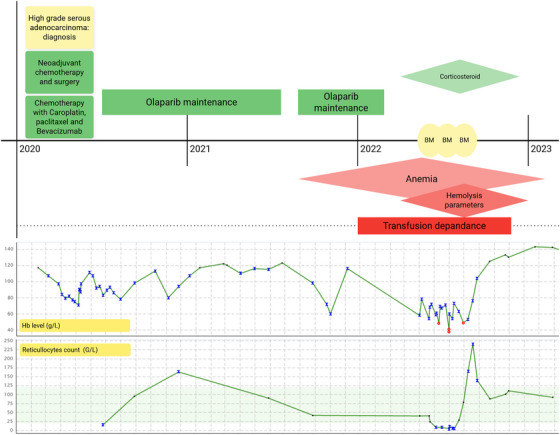
Timeline of the case with Hb and reticulocyte count evolution. BM: bone marrow; Hb: hemoglobin.

**FIGURE 2:**
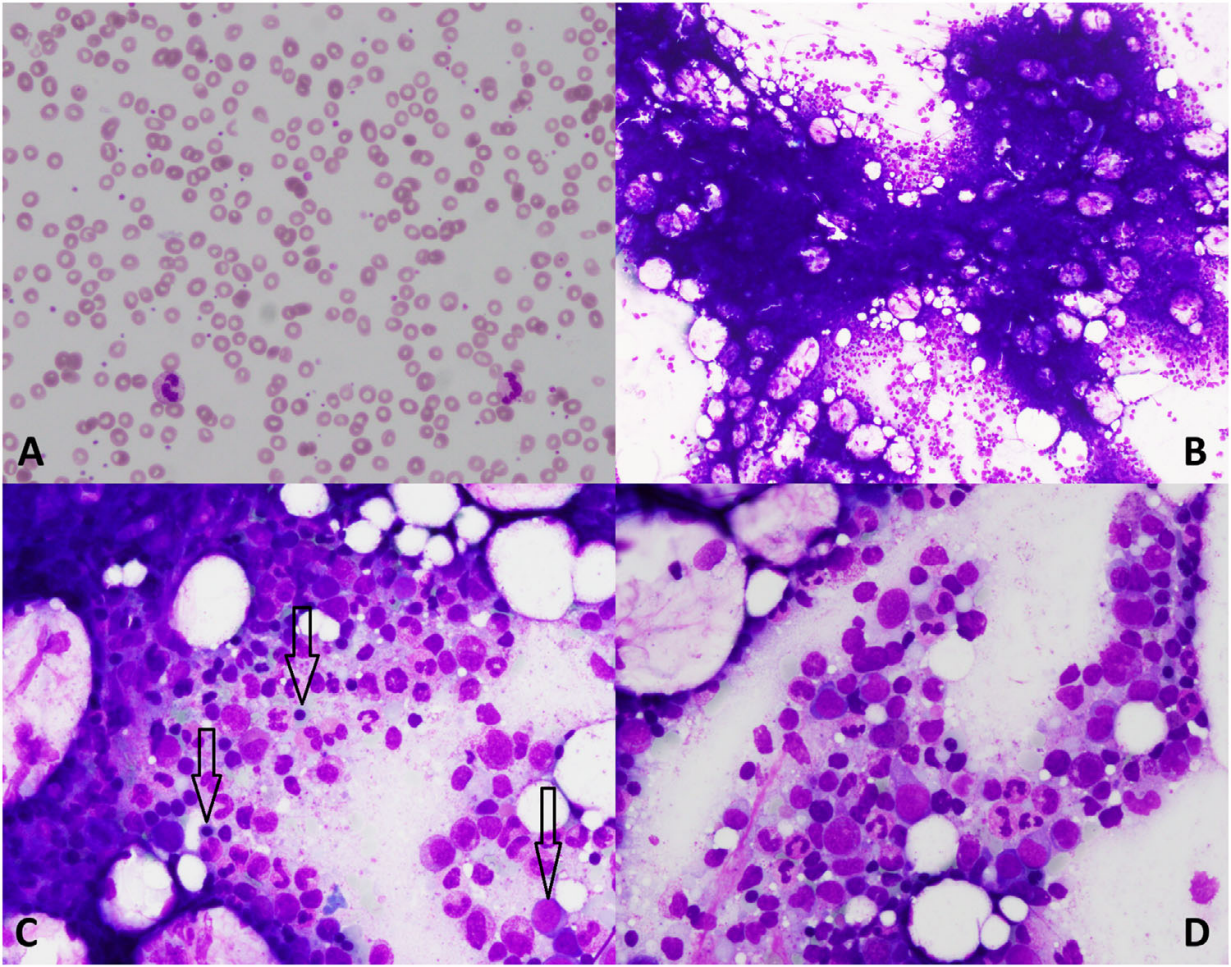
Peripheral blood smear with anispoikylocytosis, 40× magnificence (A), bone marrow aspiration image with May‐Grunwald‐Giemsa showing hypercellularity without dysplastic evidence, the granulocytic series matures up to the segmented forms, the erythroid colonies are left‐shifted, containing numerous macroblastic and megaloblastic elements but the red cell lineage matures up to the terminal forms: 10× magnificence (B) and 40× magnificence (C, D). The different stages of erythroid maturation are highlighted by black arrows.

A 71‐year‐old woman diagnosed with high‐grade serous ovarian adenocarcinoma in February 2020 was treated by six cycles of peri‐operative chemotherapy of carboplatin AUC6 Q3W and weekly paclitaxel 80 mg/m^2^, and an interval surgical debulking (Figure 1 ). The post‐operative chemotherapy was combined with bevacizumab Q3W (an anti‐VEGF inhibitor), planned for a total of 15 months. At the end of the post‐operative chemotherapy, in addition to the bevacizumab maintenance, the patient started a poly (ADP‐ribose) polymerase inhibitor, olaparib, 300 mg bid, planned for a total of 24 months. This doublet maintenance treatment was based on the cancer stage and homologous recombination deficiency (HRD) status according to the phase 3 PAOLA‐1 trial [1]. Bevacizumab was stopped 11 months later due to neurological complications. Olaparib was initiated in September 2020, 4 months after surgery, initially in concomitance with bevacizumab and continued for a total of 19 months until March 2022. Before olaparib initiation, the patient had residual post‐chemotherapy grade 1 anemia (113 g/L). After 7 months of treatment with olaparib, the patient presented a worsening hypo‐regenerative anemia, with hemoglobin (Hb) levels dropping to 54 g/L and an absolute reticulocyte count of 40 × 10^3^/µL. Due to a likely drug‐induced origin, olaparib was discontinued twice (November 2020, September 2022) for 6 weeks period, the dose was reduced to 150 mg daily since January 2021, and was definitely interrupted in March 2022. Yet anemia steadily decreased, reaching a nadir of 38/L (Grade 4). At that point, the patient did not present any bleeding, stool discoloration, dark urine, B symptoms, lymphadenopathy, bone abdominal or retrosternal pain. Of note, the patient traveled to Brazil 4 weeks before, but anemia was already present prior to this trip. The patient received a total number of 9 RBC transfusions between October 2021 and May 2022. Initial investigations ruled out iron or vitamin (folic acid, vitamin B12) deficiencies, as well as autoimmune or infectious etiologies. A potentially initial hemolytic profile with positive direct antiglobulin test (DAT) for complement (C3d) and decreased haptoglobin was noted; nevertheless, LDH was in normal range, without reticulocytosis, bilirubinemia, bilirubinuria, or urobilinogenuria. Even if olaparib had been stopped for 2 months, a further decline in reticulocytes was observed, reaching a nadir of 5×10^3^/µL in June 2022. Investigations to rule out secondary myelodysplasia (described in PARP inhibitors) due to persistent cytopenias were performed [2]. The first marrow biopsy highlighted a hypercellular marrow without dysplasia or metastatic infiltration (Figure 2), but with the presence of a clonal cytotoxic T‐large granular lymphocytic proliferation (T‐LGL) constituting 20% of cellularity, confirmed by the rearrangement of TCR. Its contribution to the current condition was uncertain as beside a hypo‐regenerative anemia. No autoimmune manifestations were initially clearly present. Next‐generation sequencing (NGS) analysis revealed a DNA methyl‐transferase 3A (DNMT3A has a myeloid epigenetic function) mutation with a variant allele frequency (VAF) of 32%. Of note, two subsequent bone marrow biopsies were non‐aspirable, however, with histology identifying multilineage dysplasia signs and intense reticuline marrow fibrosis (MF‐3) comparatively to the first one (Table 1). This feature suggests a reactive or secondary process rather than a primary myeloproliferative neoplasm, as no dysplastic megakaryocytes or JAK2/MPL/CALR mutations were reported. Such fibrosis may reflect a marrow stress potentially induced by the prolonged use of olaparib. The red blood cell lineage appeared as disorganized erythroid islands, frequently containing macroblastic or megaloblastic elements, and matured to terminal forms. However, there is no evidence supporting an erythroid maturation arrest or pure red cell aplasia (PRCA). Infectious causes (Leishmania, Babesia, CMV, EBV, parvovirus B19, tuberculosis mycobacteria, and atypical mycobacteria) were absent in marrow specimens. From the beginning of June 2022, warm autoantibodies (IgG) appeared with a highest titer of 1/512 (IgG1 and IgG3 subclasses absent) were constantly detected, with a progressive increase in LDH and bilirubin levels. At the same time, reticulocyte count started to recover, namely 3 months after olaparib discontinuation; the patient remained nevertheless transfusion dependent. High‐dose corticosteroids were therefore initiated (prednisone), initially a dose of 1 mg/kg with progressive dose reduction until discontinuation after 2 months, leading to reticulocytes increase (up to 240 g/L) and the restoration of normal hemoglobin levels (130 g/L) (Figure 1). Consequently, we concluded to a mixed etiology severe anemia, initially hypo regenerative due to prolonged PARP inhibitor toxicity, and secondly complicated with an extravascular hemolysis due to warm autoimmune hemolytic anemia (wAIHA).

**TABLE 1:** Bone marrow evolution across the different bone marrow aspirates and biopsies (information based on cytology, histology, molecular biology, and NGS).

	**1**	**2 (+2 Months)**	**3 (+3 Months)**
**Cellularity**	Increased cellularity (60%)	Increased cellularity (80%)	Increased cellularity (80%)
**Blast excess**	No	No	No
**Dysplasia**	No	Yes	Yes
**Red blood cell lineage**	The erythroid colonies are left‐shifted, containing numerous macroblastic and megaloblastic elements; the red cell lineage matures up to the terminal forms	The red cell lineage is present as disorganized erythroid islands containing frequently macroblastic, sometimes even megaloblastic elements, maturing up to terminal forms	The red cell lineage is present as disorganized erythroid islands containing frequently macroblastic, sometimes even megaloblastic elements, maturing up to terminal forms
**T‐LGL infiltration**	20%	20%	20%
**TCR monoclonality (by PCR)**	Present	Present	Present
**Fibrosis**	Traces of reticulin fibrosis on silver staining (MF 0–1)	Marked reticulin fibrosis (MF‐3)	Marked reticulin fibrosis (MF‐3)
**DNMT3A mutation by NGS**	32% VAF	Not performed	Not performed

Abbreviations: NGS, next‐generation sequencing; PCR, polymerase chain reaction; T‐LGL, T‐large granular lymphocytic leukemia; VAF, variant allele frequency.

Severe anemia due to olaparib and other PARP inhibitors is well‐documented [3, 4]. However, published studies refer to a hematologic improvement within 4 weeks post‐cessation [5]. Olaparib's prolonged toxicity (>4 weeks, nearly 10 weeks) with severe reticulocytopenia is unusual according to the literature. Studies indicate PARP‐2's critical role in erythropoiesis, particularly in hemolytic cases due to ineffective progenitor production with trials mentioning even aplastic anemia induced by olaparib [1, 6, 7]. Another explanation to the prolonged PARP toxicity may be attributed to the DNMT3A mutation. Studies in leukemia cell lines found an increased sensitivity in DNMT3A mutant cells with elevated DNA damage and already impaired PARP1 recruitment. Additional inhibition to an already PARP1‐impaired cell may contribute to sustained toxicity [8]. The simultaneous presence of dysplasia described at the medullary level in follow‐up bone marrow samples, hypo‐regenerative anemia, and an associated mutation could also suggest a myelodysplastic syndrome. However, the favorable response to prednisone alone and the complete resolution of the anemia argue against this diagnosis. For this reason, the detection of a DNMT3A mutation with a VAF of 32% is consistent with clonal hematopoiesis of indeterminate potential (CHIP), which could have initially emerged through clone selection during cytotoxic therapy, as it is known that CH clones are more resistant to proinflammatory environment [9]. However, that same CHIP clone may have also led to the increased vulnerability to PARP inhibition, as explained before. We did not consider follow‐up bone marrow aspirations necessary in the absence of anemia recurrence or the appearance of a new cytopenia.

Regarding hemolytic anemia, it could be idiopathic or secondary related to the marrow's cytotoxic T‐LGL clone. Relatively, few cases of T‐LGL proliferation are reported with an isolated hemolytic presentation [10, 11]. Although TCR gene rearrangement confirmed that clonality and the marrow infiltration reached 20% of cellularity, the absence of cytopenias involving other lineages (notably neutropenia), lack of other associated autoimmune manifestations at diagnosis, and no splenomegaly or systemic symptoms argued against a definitive diagnosis of T‐LGL leukemia. Moreover, the anemia improved significantly with corticosteroid therapy without requiring immunosuppressive agents typically used for T‐LGL leukemia, further supporting a reactive clonal expansion rather than overt leukemia.

Despite the clinical and biological improvements, we recommend regular clinical and biological follow‐up due to the presence of a monoclonal T‐LGL proliferation. Persistent DAT positivity without hemolysis signs requires no follow‐up.

This case highlights the diagnostic challenge due to the concomitant existence of a toxic and an autoimmune hemolytic anemia. Prolonged drug toxicity and marrow hypocellularity masked the hemolysis pattern. Toxic central anemia is well described, as erythropoiesis is normally sustained by PARP1 activation and is also activated by erythropoietin [12]; autoimmune features are also known in case of T‐LGL proliferation. This case highlights the necessity of a thorough evaluation in case of iPARP‐induced cytopenia in oncologic patients, especially those treated with drug with known hematotoxic adverse effects and potentially protracted evolution.

## Author Contributions

**Julien Dereme**: writing – original draft, writing – review and editing. **Konstantinos Asonitis**: writing – review and editing. **Francesco Grandoni**: writing – review and editing. **Aikaterini Liapi**: writing – review and editing. **Apostolos Sarivalasis**: writing – review and editing. **Gerasimos Tsilimidos**: writing – original draft, writing – review and editing.

## Ethics Statement

Informed consent was obtained from the patient.

## Conflicts of Interest

The authors declare no conflicts of interest.

## Clinical Trial Registration

The authors have confirmed clinical trial registration is not needed for this submission

## Data Availability

The authors have nothing to report.
